# How Body Balance Influences Political Party Evaluations: A Wii Balance Board Study

**DOI:** 10.3389/fpsyg.2012.00536

**Published:** 2012-12-07

**Authors:** Katinka Dijkstra, Anita Eerland, Josjan Zijlmans, Lysanne S. Post

**Affiliations:** ^1^Institute of Psychology, Erasmus UniversityRotterdam, Netherlands

**Keywords:** embodied cognition, conceptual metaphors

## Abstract

Embodied cognition research has shown how actions or body positions may affect cognitive processes, such as autobiographical memory retrieval or judgments. The present study examined the role of body balance (to the left or the right) in participants on their attributions to political parties. Participants thought they stood upright on a Wii^™^ Balance Board, while they were actually slightly tilted to the left or the right. Participants then ascribed fairly general political statements to one of 10 political parties that are represented in the Dutch House of Representatives. Results showed a significant interaction of congruent leaning direction with left- or right-wing party attribution. When the same analyses were performed with the political parties being divided into affiliations to the right, center, and left based on participants’ personal opinions rather than a ruling classification, no effects were found. The study provides evidence that conceptual metaphors are activated by manipulating body balance implicitly. Moreover, people’s judgments may be colored by seemingly trivial circumstances such as standing slightly out of balance.

## Introduction

What happens to our thoughts if we reach up or down, lean to the left or the right? For many common experiences, such as reaching up for a glass high in a cupboard, or leaning sideways to grab a leaflet, no major thoughts will come to mind during these routine and goal-specific physical actions. There are circumstances, however, in which body position or body movements may facilitate access to stored information or activate implicit or explicit notions about more abstract concepts of emotions, power, or politics.

Several studies provide support for this assumption by demonstrating facilitation of autobiographical memory retrieval following specific body movements or body position. One study (Dijkstra et al., 2007) showed how assuming a congruent body position during autobiographical memory retrieval with the body position of the original experience (i.e., lying down in a reclined position while retrieving a memory about visiting the dentist) resulted in faster retrieval times compared to an incongruent body position (i.e., standing in a jumping-jack position, while retrieving a memory about a visit to the dentist).

Another study looked at the nature and efficiency of autobiographical memory retrieval after participants moved marbles upward or downward while thinking about personal events from the past (Casasanto and Dijkstra, 2010). When participants retrieved memories to positive and negative prompts when they moved marbles upward and downward, they did this faster under congruent valence-direction conditions (positive memories and upward movements and negative memories with downward movements) than under incongruent conditions. They must have implicitly activated the “positive is up and negative is down metaphor” for no facilitation of congruent movement-valence combinations would have been found otherwise.

The results of these studies suggest an important linkage of bodily action, memory, and emotion. Physical actions can have an impact on how and what we think and facilitate access to earlier experiences. This supports the idea that movements may activate more abstract concepts that have a basis in our own experiences, which is also consistent with theories on metaphorical mental representations.

According to the Conceptual Metaphor Theory (Lakoff and Johnson, 1980), abstract concepts are understood in terms of concrete concepts and experiences (e.g., Lakoff, 1987). People tend to use concrete concepts, such as “up” and “down” in space, to talk about more abstract concepts, in this case “power.” As each metaphor only defines specific aspects of an abstract concept, abstract concepts are defined in more than one concrete concept. For example, “love” may be represented in terms of both “warmth” and “closeness.” Conversely, “warmth” may also be considered in the attribution of the abstract concept “anger” and “up” may be a representation of both power and (positive) emotion. Moreover, metaphorical concepts arise from physical and cultural experiences. We cheer with our arms up when our favorite team wins a championship and point our thumbs down when a referee makes a bad decision. These are instances of shared experiences that eventually shape the representation of the concept. These concepts and representations not only emerge from our physical experiences but from dominating cultural conventions as well (Lakoff and Johnson, 1980). The positive connotation of the thumbs-up gesture, for example, is not universal across cultures, but denotes a sexual insult in South-American culture.

A common type of metaphorical concept is the orientational metaphor that represents concepts in a linear manner (Lakoff and Johnson, 1980). Examples are the up or down metaphor (up is power, positive, more), and the left or right metaphor (right is more, future, conservative). A difference between the up-down metaphor and the left-right metaphor is that the former has an experiential basis when it reflects power (a child always looks up to an adult) or valence (cheering is up), whereas the left-right orientation is based on conventions such as the mental number line and the organization of political parties on a left-right dimension. The mental number line theory posits that smaller magnitudes are associated with a location on the left and larger magnitudes on the right of this line (Restle, 1970). Research has empirically supported this notion (Dehaene et al., 1993; Eerland et al., 2011).

The left-right dimension in politics originates from the spatial organization of the French Legislative Assembly of conservatives on the right and liberals on the left (Goodsell, 1988). If abstract concepts are activated as a result of concept-matching concrete experiences, it follows that left/right-manipulations of bodily actions can activate the left/right metaphor associated with politics and consequently influence one’s thinking about politics. Several instances of such activations have been demonstrated in studies from different countries with a differential representation of political parties.

Oppenheimer and Trail (2010) addressed the question whether physical stimuli activating spatial concepts could influence political judgments. Using a right/left metaphor to distinguish between conservatives on the right and liberals on the left in the US could possibly help individuals’ understanding of the political landscape. Support for this notion of political spatial representation comes from web pages that contain more frequently left-Democrat and right-Republican associations than the other way round. Three experiments were conducted to demonstrate the activation of a political concept based on a spatial manipulation.

In experiment 1, participants were asked to squeeze a clothespin shaped hand-grip to a closing position for 5 s with either the right or left hand. After that, they were asked to what extent they agreed with Democrats and Republicans on political issues on an eight-point scale. Handedness and political affiliation were recorded as well. The results indicated a significant interaction between grip-hand and political agreement with participants squeezing with their left hand agreeing more with Democrats than participants who squeezed with their right hand. The second experiment manipulated spatial orientation differently by having participants sit in a chair that tilted to the right or the left after a wheel was removed. Participants answered the same questions as in the first experiment. Again an interaction was shown. Participants who leaned to the left were more likely to agree with Democrats. A third experiment was conducted online to recruit more Republicans. Here, participants made responses with a mouse to a visual target on the left or right screen. Again, only an effect for the left manipulation and stronger agreement with Democrats was found. Although consistent results were demonstrated for endorsement of Democrats after left-manipulations, no effects were found for the right-manipulations, which limits the generalizability of the results. Moreover, spatial manipulations varied in subtlety which makes it difficult to draw strong conclusions regarding the efficacy of the manipulations tested. Also, endorsement of political attitudes was assessed with two questions on a rating scale only. The validity of the dependent measures may therefore be questionable.

Another study examined the effect of left-right manipulations on the activation of political concepts in a country that is represented by 10 political parties in the first and second Chamber and is governed by a coalition of parties, Netherlands (van Elk et al., 2010). In the Dutch political system, political parties can be considered left-wing, right-wing, and central. A consequence of this system is that voters on one party may not always see the party issues being followed-through because of coalition agreements that necessitated compromises on certain issues. The large number of parties and a level of uncertainty regarding adherence to certain issues may affect the activation of abstract political concepts based on spatial (left-right) manipulations.

The study examined the speed of processing acronyms referring to names of political parties on co-activation of spatial associations (van Elk et al., 2010). Additionally, the role of one’s own political preference was taken into account. It was expected that spatial associations should hold irrespective of one’s own standpoint. In four experiments, participants made categorization responses to acronyms that represented names of political parties or names of public broadcasting companies by pressing either a button with the right or the left hand following a cue that indicated with which hand they should respond (< for left and > for right). Response facilitation was expected when participants responded with the hand that was congruent (left or right) with the perceived orientation of the political party (left-right).

The results for the experiments indicated a significant party (left-right) by action (left-right) interaction as predicted. In experiment 1, right hand responses were faster for right-wing parties but no differences for left-wing parties occurred. In experiments 2 and 3, the opposite pattern was found, whereas the results for experiment 4 mimicked those of experiment 1. Although the results demonstrated that processing acronyms of political parties is associated with implicit activation of spatial associations, no consistency with regard to the left or right manipulation was found. Moreover, participants with a preference for the political right showed a stronger effect size for left-wing parties, possibly because this (minority) group perceived the distance of left-wing parties to be larger as a result of their own political orientation.

We can conclude that both studies (Oppenheimer and Trail, 2010; van Elk et al., 2010) demonstrate an activation of political concepts based on spatial manipulations. These results are in line with the co-occurrence of references to political parties and spatial orientations in the public debate, news broadcasts, social interactions, and online guidance tools for political orientation (Landauer et al., 1998). A remaining question and a possible weakness of these studies is that none of the experiments demonstrated an effect of both the left and right manipulation on the activation of political concepts. The relatively low number of participants with a right-wing political orientation may have played a role, but does not explain the differential outcomes in the van Elk et al. (2010) studies.

In contrast to the Oppenheimer and Trail study, the current study employed an implicit left-right balance manipulation. Participants were not aware of the fact that their balance was manipulated. Rather, they were under the impression that they were standing straight up. Balance manipulation was used in combination with stimuli materials that were general and would not automatically activate a political party. Political statements were generated that did not obviously belong to a left-wing or right-wing party. A certain level of ambiguity was expected to be more vulnerable to the effects of the body balance manipulation. In addition, more specific questions regarding political knowledge and orientation were asked. The prediction was that participants who were manipulated to lean to the left or the right were more likely to ascribe political statements to left-wing or right-wing political parties.

## Materials and Methods

### Pilot study

A pilot study was conducted on nine volunteers to test political statements with regard to the ascribed political orientation of the statements (left/right) and political affiliation (name of the party). These political statements were taken from political party programs and reworded in more general terms to make them less obvious as coming from a specific political party. The statements were considered left-wing by about half participants and right-wing by the other half. Statements that were considered left-wing or right-wing by (almost) all participants were eliminated or reworded. None of the 32 statements were attributed to the same political party by all participants. All raters considered the task moderately difficult (mean of 3 on a five-point rating scale) and all of the statements were considered more or less equally difficult to rate (mean of 3 on a five-point scale). Together, the ratings confirmed that the statements were general and difficult enough to create uncertainty with regard to what party it should be attributed to.

### Current study

#### Participants

A total of 32 participants took part in the experiment (mean age = 20.29, SD = 2.16 range = 18–26), 94% women, 85% right-handed. Data from four participants had to be removed because of procedural difficulties (no native speaker of Dutch, too much movement during the experiment), or lack of familiarity with the stimuli materials. The remaining participants had a rather low interest in politics (an average of 2.5 cm, SD = 2.1 on a 10 cm VAS scale). Their knowledge of politics was also rather low (an average of 2.4 cm SD = 1.8, on a 10 cm VAS scale). Only 68% of the participants had voted in the past but this relatively low number can be partly due to the number of 18-year olds (21%) who may have been too young to vote during the elections last year. Overall, participants can be considered moderately involved in politics. The experiment complied with the regulatory standards of the psychology department’s ethics committee. Participants consented to their involvement in the experiment prior to their participation and were informed about the procedure of the study.

#### Stimuli materials

Thirty-two political statements from the pilot study were used for the current study. An overview of these statements with the instructions for participants is listed in Appendix A. Fillers were 32 statements regarding well known television programs from public broadcasting companies. These statements referred to the title, content, or presenter of these programs. A randomized order of political statements and fillers was created with the restriction that no more than two political statements or fillers would follow one another.

#### Apparatus

A Nintendo Wii balance board was used to manipulate body position. Recently, Wii balance boards have been used in experimental studies (Clark et al., 2010; Eerland et al., 2011) and demonstrated good psychometric properties. Center of Pressure (COP) can be quantified reliably with a balance board and has shown very good test-reliability on several different test protocols. In other words, the Wii balance board can be considered as a valid tool for assessing balance (Clark et al., 2010).

#### Procedure

Upon entering the lab, participants removed their shoes and had their height recorded. Based on their height, the position of the computer screen was adjusted so that no changes in posture would occur as a result of reaching or bending to see the text on the screen. Next, they stepped on a Wii balance board for calibration and manipulation of body position. First, the COP was calibrated, then their posture was manipulated such that participants thought that their COP was in the middle of the balance board even though it was manipulated to the right or the left. This manipulation was very subtle (about a 2% change in weight proportion on left and right sensors of the board) and never noticed by participants. To ensure maintenance of the manipulated body position, their COP was displayed throughout the experiment on a computer screen as a square within a surrounding circle. If they strayed from this circle, a warning signal occurred to prompt them to retake their neutral COP position. In reality, the signal occurred to keep participants in the left or right body position.

After calibration, participants responded to statements that appeared on a screen displayed above a fixation circle that either concerned statements from political parties or television programs. When political statements were presented, participants were asked to name the Dutch political party they attributed the statement to. They could choose from 10 political parties that were in the Dutch House of Representatives at that time. Based on an existing division of the parties in a left/right horizontal axis and a progressive/conservative vertical dimension grid (Kieskompas Tweede Kamerverkiezingen, 2010, see Figure 2) five left-wing (progressive), four right-wing parties (conservative), and one center party could be identified (see Figure 3 for the grid). Statements with descriptions of television shows were included to mask the true purpose of the experiment. For this filler task, participants had to name the broadcasting association (from a total of nine) that produced that particular program. A sheet with the acronyms of the names of these broadcasting companies listed vertically and in alphabetical order was taped onto the computer desk to ensure that participants knew the names. This focus on the broadcasting companies was meant to take away attention from the body position/political party manipulation. Participants were instructed to come up with a party/broadcasting company even if they were uncertain or did not know the answer. Participants were led to believe that the computer registered the answers, even though in truth, the experimenter wrote down the answers given by the participants and moved the statements forward with a Wii-mote. This way, participants would not be tempted to turn toward the experimenter to provide the answers which would possibly influence their body position.

Halfway through the experiment, after responding to 32 political/television statements participants played a balance game, supposedly to get a break from the task, but in reality to change body position (from left-to-right or vice versa). Body position was counterbalanced across participants (left position first or right position first), and so was the order of statements (half of the participants received the statements in reverse order). Afterward, participants answered questions regarding their political knowledge, political interest, and filled in the parties and their own political affiliation on the progressive/conservative and left/right axes of the grid.

## Results

The main prediction was that participants would attribute more political statements to right-wing political parties when leaning to the right than when leaning to the left. For participants leaning to the left, the opposite effect was predicted: higher attribution of political statements to left-wing when leaning to the left, than when leaning to the right. Given that only one political party qualified as a center party, the analysis was limited to attributions to left or right-wing parties^1^. Table 1 presents the proportions and standard errors of the relevant variables.

**Table 1:** **Mean proportions, standard errors (SE), and lower-and upperbound attributions to political parties**.

	Left-wing attribution	Center attribution	Right-wing attribution
Leaning left	0.43 (0.03) 0.36–0.49	11 (0.02) 0.06–0.16	0.46 (0.03) 0.40–0.52
Leaning right	0.37 (0.03) 0.31–0.42	0.10 (0.02) 0.06–0.14	0.54 (0.03) 0.48–0.60

**Figure 1:**
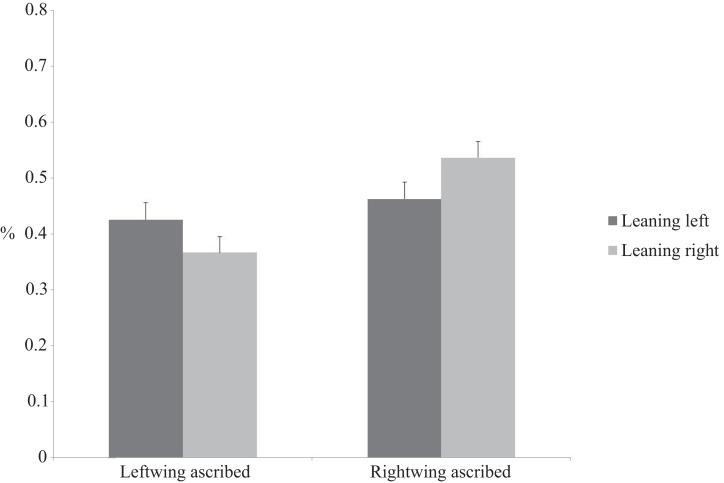
**Number of ascribed statements to left- and right-wing parties by leaning position**.

A two (leaning direction: left vs. right) by two (political attribution: left vs. right) repeated measures ANOVA on the proportions of answers demonstrated a main effect of attribution, *F*(1,27) = 4.80, *p *< 0.05, η^2^ = 0.151, and a leaning direction by attribution interaction, *F*(1,27) = 6.29, *p* < 0.05, η^2^ = 0.189. Figure 1 displays the results. Subsequent simple effects analyses demonstrated that when leaning to the right participants ascribed more political statements to right-wing political parties than when leaning to the left, *F*(1,27) = 7.94, *p* < 0.01, η^2^ = 0.17. When participants were leaning to the left they tended to ascribe more political statements to left-wing political parties than when leaning to the right, though this effect showed only a trend toward significance, *F*(1,27) = 3.06, *p* = 0.07, η^2^ = 0.12.

**Figure 2:**
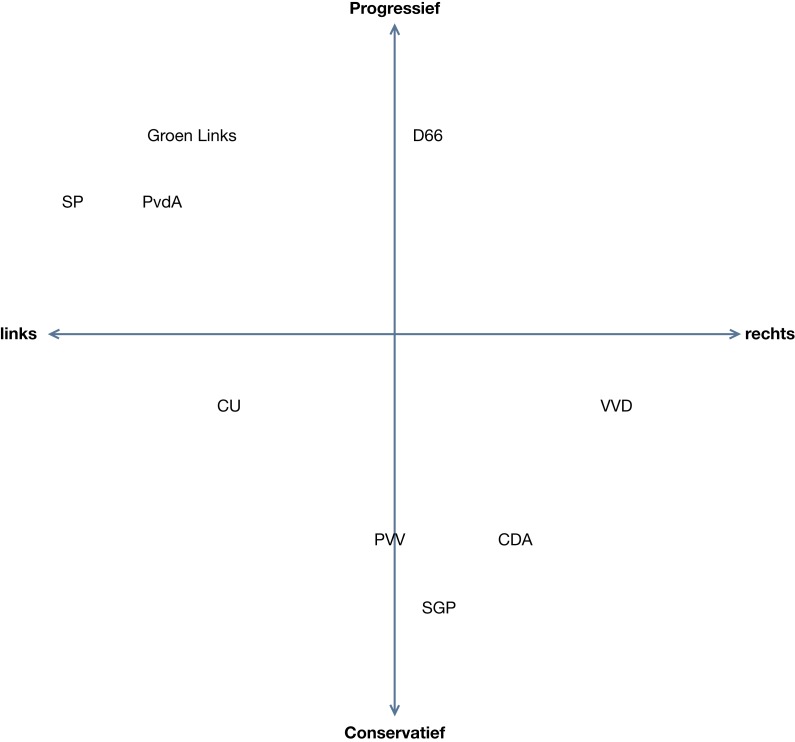
**Overview of political parties on the grid for the general elections 2010 in the Netherlands**.

**Figure 3:**
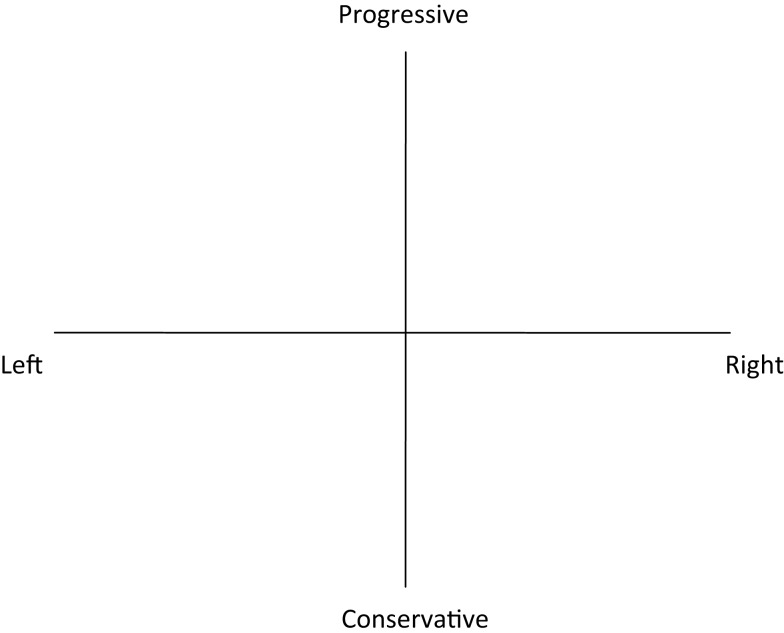
**Place the political parties listed below on the correct location in the grid**. Then position your own political affiliation in the grid. The political parties to be placed in the grid are: CDA, CU, D’66, GL, PvdA, PVV, SGP, SP en VVD.

The same analysis was performed with the political parties being divided into right and left based on participants’ personal opinions on what political parties’ position should be on the grid, rather than on their actual political positions on the grid. No effects were found (*p* > 0.05). A similar analysis was conducted with the addition of their political affiliation as a between subjects factor based on how participants positioned themselves on the grid. No effects were found either (*p* > 0.05).

Participants demonstrated poor knowledge on how parties are organized along left-right and conservative-progressive dimensions. On average, participants were able to place less than half the parties correctly on the grid in the left-right dimension (*M* = 4.57, SD = 1.67, range = 1–7). A sample of 33 comparable participants that attributed political parties only on a left-right dimension showed a much higher correct attribution of political parties (*M* = 5.97, SE = 0.18) than the current sample (*M* = 4.57, SE = 0.33).

## Discussion

The results support the prediction that conceptual metaphors are activated when body balance is manipulated. People’s judgments appear indeed to be affected by seemingly inconsequential circumstances such as leaning slightly to the left or right on a Wii balance board. In contrast to earlier studies, this manipulation was implicit and unknown to the participants involved. In some of the experiments in the Oppenheimer and Trail (2010) study and the van Elk et al. (2010) study, participants could have been aware of the left-right manipulation. The implicit manipulation and the generality and difficulty of the political statements seem to have been effective.

The marginal effect of the manipulation to the left, reminds us of the earlier studies that also did not demonstrate effects of the manipulation to both sides in one experiment. This outcome cannot be attributed to the design of these studies. Some of the experiments had a between subjects design and some had a within subjects design, just like the current study. Moreover, counterbalancing prevents possible effects from fatigue. Possibly, with more statements being attributed to right-wing than left-wing parties fewer statements were available for attribution to left-wing parties.

Surprisingly, participants’ own political affiliation or personal organization of political parties on the grid did not matter. Scoring these parties on horizontal and vertical dimensions may have been difficult because of the joint left-to-right and progressive-conservative axes. The grid may not have been the right task to use for this purpose. So far, it has mostly been applied as an online task to determine party preference as an aid to prospective voters. Positioning of parties on the grid as outcome in the party preference process depends on which issues are prioritized by the person taking the test. This adds to the complexity of the task. Moreover, participants in the current sample may not have been motivated to fill in the grid given their low-to-moderate scores on political knowledge and interest.

The study contributes to the current discussion in the field of embodied cognition and Conceptual Metaphor Theory. Abstract concepts seem grounded in concrete experiences, even if these experiences are based on conventions (left-right = left-wing and right-wing). Subtle manipulations in combination with general stimuli materials may be an effective way to demonstrate such activations. The role of the body on cognitive processes has again been demonstrated and for a somewhat different task as before: attribution of political parties based on statements rather than reaction times in a go, no-go task (van Elk et al., 2010), or endorsement of political parties (Oppenheimer and Trail, 2010). The outcomes contribute to a growing body of evidence suggesting that cognition is grounded in action, even with subtle actions.

Future studies could assess whether the activation of abstract concepts is more effective when the whole body is involved, as was demonstrated in this study, or when parts of the body are involved (van Elk et al., 2010). If the body is considered essential for cognitive reactions to occur as is proposed by the strong view of embodiment, manipulations with the whole body may be more effective than with parts of the body. On the other hand, if the body is merely a tool in this respect, manipulations with part(s) of the body may be equally effective.

Another issue is the bi-directionality of these phenomena (Rueschemeyer et al., 2009; Miles et al., 2010a). Miles et al. (2010b) showed that direction of apparent motion in the form of dots appearing to move toward or away from the center of a display affected their mental time travel. Perceived backward motion was associated with thoughts about the past whereas perceived forward motion was associated with future-oriented thoughts. An earlier study by Miles et al. (2010a) had showed a converse relationship, that of moving forward or backward as a result of mental time travel to the future or the past. It is plausible then that after demonstrating an effect of bodily actions on the cognitive process of attribution, an opposite effect could be demonstrated as well. It is not unlikely that if participants are primed with a left-wing or right-wing political orientation, that they may unconsciously start leaning to the left or the right. If so, bi-directional processes would be at play.

What should be done during elections? Making sure that voters sit and are unable to lean to the right or the left? Such precautions are probably not necessary. Most effects that have been documented in embodied cognition research are short-lived, context-dependent, and only appear after very specific and scope-limited manipulations and seem to affect reaction times, ratings and attributions to parties, not the selection of a party to vote for. Otherwise, we could never be in a warm room without worrying that we may feel affective or hostile toward another person in the room or feel miserable whenever we are shorter or at the bottom of a slope because we lack power.

## Conflict of Interest Statement

The authors declare that the research was conducted in the absence of any commercial or financial relationships that could be construed as a potential conflict of interest.

## Footnotes

^1^An analysis including the center party yielded the same results as the analysis without the center party. In both leaning conditions, the same number of statements were attributed to the center party. Posture had no effect on attribution to center parties.

## Appendix A

Read the statement and tell me to what political party the statement should be ascribed:

A dedicated group of police officers should be assigned to only focus on dealing with sexual offenses.
Fired employes should be able to claim supplementary training.
Future generations should not be left with the debt of the crisis.
Teachers in primary and secondary education should attend refresher courses on a regular basis.
There should be a law to determine benefits after job termination.
There should be local hotlines for nuisance in and around residential dwellings.
There should be one national general inspection and audit for entrepreneurs.
Organizers of sport events should pay for the deployment of police officers themselves.
The number of available rental homes should increase gradually.
As soon as someone turns 65, this person has the right to retire.
When signing with a health care insurance company existing handicaps should be taken into account.
Gifted children should be given the opportunity to receive an alternative education program.
The Dutch government should follow the European policy regarding regulations around fishery.
More money should become available for caregivers.
Everyone below the age of 23 has a mandatory job or school education.
To resolve issues related to traffic jams, the government should invest in better public transport.
Human trafficking should be handled by specialized judges.
The European Union is very important for welfare in the Netherlands.
There should be clear guidelines regarding the provision of hard drugs.
There should be mandatory parenting support for families dealing with problems.
Civilians should pay administrative costs when they complain about the police.
Norms should be set up to determine the distance from every house to the nearest public transport stop.
The number of public broadcasting stations should be reduced in order to reduce government spending.
Reclassification of local governments is necessary to increase administrative power.
The government should be allowed to take extra security measures in order to fight crime.
Rules regarding road safety should be tightened.
Parents should not receive child support if their child skips school on a regular basis.
European cooperation is needed to counteract terrorism.
The Netherlands should no longer invest in organic farming.
People immigrating to the Netherlands should be helped more extensively concerning integration.
The chronically ill and their families should receive more support.
The national armies of countries in the European Union should cooperate more closely.

## Appendix B

**Table:**

		Leaning left		Leaning right	
		(list 1 = group 1, list 2 = group 2)		(list 1 = group 2, list 2 = group 1)	
		Attributed left	Attributed right	Attributed left	Attributed right
List 1	Statement 1	0.500	0.357	0.357	0.357
	Statement 2	0.929	0.071	0.786	0.214
	Statement 3	0.500	0.500	0.357	0.643
	Statement 4	0.500	0.429	0.357	0.500
	Statement 5	0.429	0.500	0.429	0.429
	Statement 6	0.357	0.571	0.071	0.643
	Statement 7	0.571	0.429	0.286	0.643
	Statement 8	0.286	0.714	0.143	0.643
	Statement 9	0.429	0.571	0.357	0.571
	Statement 10	0.571	0.357	0.571	0.286
	Statement 11	0.357	0.571	0.357	0.571
	Statement 12	0.357	0.643	0.357	0.500
	Statement 13	0.357	0.643	0.214	0.714
	Statement 14	0.643	0.357	0.643	0.357
	Statement 15	0.357	0.571	0.286	0.643
	Statement 16	0.643	0.357	0.500	0.429
List 2	Statement 17	0.357	0.429	0.214	0.643
	Statement 18	0.214	0.571	0.214	0.786
	Statement 19	0.071	0.643	0.429	0.571
	Statement 20	0.643	0.286	0.500	0.429
	Statement 21	0.143	0.429	0.000	0.500
	Statement 22	0.571	0.357	0.429	0.500
	Statement 23	0.143	0.571	0.214	0.643
	Statement 24	0.214	0.571	0.500	0.500
	Statement 25	0.429	0.071	0.357	0.500
	Statement 26	0.357	0.500	0.500	0.500
	Statement 27	0.000	0.571	0.286	0.571
	Statement 28	0.286	0.571	0.143	0.643
	Statement 29	0.429	0.286	0.357	0.571
	Statement 30	0.857	0.071	0.500	0.500
	Statement 31	0.929	0.071	0.571	0.429
	Statement 32	0.000	1.000	0.286	0.643
